# The influence of ultraviolet reflectance differs between conspicuous aposematic signals in neotropical butterflies and poison frogs

**DOI:** 10.1002/ece3.7942

**Published:** 2021-09-18

**Authors:** Justin Yeager, James B. Barnett

**Affiliations:** ^1^ Biodiversidad Medio Ambiente y Salud Universidad de Las Américas Quito Ecuador; ^2^ Psychology, Neuroscience & Behaviour McMaster University Hamilton ON Canada

**Keywords:** aposematism, butterflies, coloration, Dendrobatidae, sexual signals, UV reflection, visual modeling

## Abstract

Warning signals are often characterized by highly contrasting, distinctive, and memorable colors. Greater chromatic (hue) and achromatic (brightness) contrast have both been found to contribute to greater signal efficacy, making longwave colored signals (e.g., red and yellow), that are perceived by both chromatic and achromatic visual pathways, particularly common. Conversely, shortwave colors (e.g., blue and ultraviolet) do not contribute to luminance perception yet are also commonly found in warning signals. Our understanding of the role of UV in aposematic signals is currently incomplete as UV perception is not universal, and evidence for its utility is at best mixed. We used visual modeling to quantify how UV affects signal contrast in aposematic heliconiian butterflies and poison frogs both of which reflect UV wavelengths, occupy similar habitats, and share similar classes of predators. Previous work on butterflies has found that UV reflectance does not affect predation risk but is involved in mate choice. As the butterflies, but not the frogs, have UV‐sensitive vision, the function of UV reflectance in poison frogs is currently unknown. We found that despite showing up strongly in UV photographs, UV reflectance only appreciably affected visual contrast in the butterflies. As such, these results support the notion that although UV reflectance is associated with intraspecific communication in butterflies, it appears to be nonfunctional in frogs. Consequently, our data highlight that we should be careful when assigning a selection‐based benefit to the presence of UV reflectance.

## Graphical abstract

The presence of UV aposematic signals is paradoxical as UV perception is not universal, and evidence for its utility is at best mixed. We found that UV only appreciably affected visual contrast in the butterflies. As the butterflies, but not the frogs, have UV‐sensitive vision, these results support the notion that UV reflectance is associated with intraspecific communication, but appears to be non‐functional in frogs. Consequently, we should be careful when assigning a selection‐based benefit from UV reflectance.

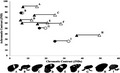

## 1 INTRODUCTION

Our contemporary understanding of the evolution of bright and conspicuous color patterns is rooted in the work of the early pioneers of evolutionary themes such as natural selection. Charles Darwin developed the theory of sexual selection to explain the presence of conspicuous ornamentation, but realized it could not account for the presence of bright colors in nonreproductive Lepidopteran larvae (Darwin, 1871). Alfred Russel Wallace, on the other hand, was skeptical of sexual selection and instead built on the work of John Jenner Weir and Henry Walter Bates, to outline a theory of aposematic signals, that was later developed further by Edward Bagnall Poulton (Caro, 2017; Caro & Ruxton, 2019; Marchant, 1916; Poulton, 1890).

Aposematic and sexually selected color patterns are highly diverse, but such signals are often characterized by high visual contrast both between pattern components within an organism, and to the background against which the organism is viewed (Andersson, 1994; Ruxton et al., 2019; Stevens & Ruxton, 2012). Brighter and more conspicuous signals are commonly associated with more potent defenses and greater reproductive fitness, such that predators are more easily deterred (Aronsson & Gamberale‐Stille, 2008; Forsman & Herrström, 2004; Forsman & Merilaita, 1999; Halpin et al., 2020; Prudic et al., 2006; Stevens et al., 2010), rivals are more wary, and potential mates more interested when signals are highly contrasting (Andersson, 1994; Endler, 1983; Ryan & Keddy‐Hector, 1992; Svensson & Wong, 2011).

High signal contrast can be achieved via two interconnected visual pathways: achromatic contrast (luminance/brightness) and chromatic contrast (hue/saturation). In vertebrates, achromatic contrast is measured as a single intensity value received by longwave sensitive photoreceptors, whereas hue is perceived through opponent processing by two or more photoreceptors that differ in their peak wavelength sensitivity (Kelber & Osorio, 2010; Vorobyev & Osorio, 1998). Consequently, different colors contribute to visual contrast in different ways: Longwave colors (e.g., red, orange, and yellow) contribute to both achromatic and chromatic contrast, whereas shortwave colors (e.g., blue and ultraviolet (UV)) only significantly affect chromatic contrast (Stevens & Ruxton, 2012; Umbers, 2013).

For this reason, conspicuous signals frequently generate high visual contrast by combining bright long wavelength colors with low luminance black (Stevens & Ruxton, 2012). Short wavelength colors, including UV, can also create high contrast and are occasionally incorporated into seemingly conspicuous signals (Umbers, 2013). However, evidence for the efficacy of UV in aposematic signals has been mixed, with no compelling confirmation that naturally occurring UV signals are effective at deterring predators despite some evidence that UV signals can be learned (Lyytinen et al., 2001; Werner et al., 2012, 2014, 2014). Moreover, rather than preventing attacks UV‐containing (UV+) signals can instead deflect attacks to more expendable body parts (Olofsson et al., 2010), or they may actually increase predation risk (Lyytinen et al., 2004). Despite the discovery of UV reflectance attracting much attention, perhaps due to our own inability to perceive such signals, we currently lack a complete understanding of if, or to what extent, UV reflectance contributes to aposematic signaling.

In the Neotropics two independent, and completely unrelated, radiations of bright conspicuous colors have drawn much scientific attention: the heliconiian butterflies (Heliconiinae; Nymphalidae) and the poison frogs (Dendrobatidae: Anura). Both groups are found in similar rainforest habitats, are highly toxic, are at risk from similar predatory taxa, and have become renowned for their high diversity of species and bright colors (Merrill et al., 2015; Stynoski et al., 2015), including the presence of UV in their conspicuous signals (Briscoe et al., 2010; Yeager & Barnett, 2020).

The heliconiian butterflies, especially the specious genus *Heliconius*, have been extensively studied in relation to color patterns that both warn predators of their potent toxins and signal important identifying information to conspecifics (Merrill et al., 2015). Complex mimicry systems have evolved to exploit predator avoidance learning, and subtle visual cues are used by conspecifics to identify potential mates (Bybee et al., 2012; Dell'Aglio et al., 2018; Merrill et al., 2015). The butterflies are potential prey to a diversity of predators, including insectivorous birds and lizards, many of which have vision sensitive to UV reflectance (Dell'Aglio et al., 2018). Similarly, the butterflies themselves are also able to see ultraviolet with some species having evolved two UV‐sensitive photoreceptors that allow for fine‐scale discrimination of UV wavelengths (Briscoe et al., 2010; Finkbeiner & Briscoe, 2020). For example, female *Heliconius erato* have a functionally pentachromatic (cone peak sensitivities (λ_max_) of 355 nm, 390 nm, 470 nm, 555 nm, and 600 nm) visual system (McCulloch et al., 2016). The evolution of duplicate UV‐sensitive opsins is constrained to the genus *Heliconius* and appears to have co‐evolved with the presence of specific UV‐reflecting yellow pigments which are predicted to be important in directing social behaviors between both conspecifics and heterospecifics (Briscoe et al., 2010).

Poison frogs have similarly become a model system for understanding the interplay between aposematic and sexually selected signals (Stynoski et al., 2015). Brighter and more contrasting colors offer greater protection from predators (Dreher et al., 2015; Maan & Cummings, 2012), are more intimidating to rivals (Crothers & Cummings, 2015; Crothers et al., 2011; Galeano & Harms, 2016), and are favored by potential mates (Dreher et al., 2017; Maan & Cummings, 2008; Maan & Cummings, 2009). Poison frogs, like heliconiian butterflies, are at risk from a wide range of UV‐sensitive predators, including birds, snakes, and lizards (Alvarado et al., 2013; Dreher et al., 2015; de Lanuza & Font, 2014; Lenger et al., 2014; Maan & Cummings, 2012; Master, 1998; Santos & Cannatella, 2011; Saporito et al., 2007; Siddiqi et al., 2004; Willink et al., 2013). However, unlike the butterflies, poison frogs are not known to possess UV‐sensitive vision. The one well‐described poison frog visual system being that of *Oophaga pumilio*, which has trichromatic vision (λ_max_ of 466 nm, 489 nm, and 561 nm) that both lacks a UV‐sensitive cone and has a lens that filters out UV wavelengths (Siddiqi et al., 2004; Yovanovich et al., 2020).

Despite many similarities in color diversity, chemical defense, the visual environment, and the predator community, ultraviolet reflective colors are relatively common in heliconiian butterflies but seemingly rare in poison frogs (Briscoe et al., 2010; Bybee et al., 2012; Yeager & Barnett, 2020). Indeed, we recently described the first example of UV reflectance in poison frogs, from an Ecuadorian population of *Oophaga sylvatica*. We found that although UV showed up brightly in photographs, it added little to internal color pattern contrast in this population (Yeager & Barnett, 2020). Here, we expand these previous findings to describe UV reflectance in two more species of poison frog (*Ameerega bilinguis* and *Epipedobates tricolor*). We compare the contribution of UV to signal contrast between these two dendrobatid frogs and five species of heliconiian butterflies and then discuss the importance of predator versus conspecific vision in light of an extensive literature on heliconiian butterflies, to point to potential explanations for the evolution of UV reflectance in these frogs.

## 2 METHODS

### 2.1 Photography

We photographed two species of Neotropical poison frog (*Ameerega bilinguis* and *Epipedobates tricolor* “Cielito” morph, Dendrobatidae) and five species of Neotropical aposematic butterfly (*Eueides isabella*, *Heliconius atthis*, *H. erato*, *H. ismenius*, and two subspecies of *H. melpomene*, Nymphalidae). The frogs (*A. bilinguis* = 5; *E. tricolor* = 4) were photographed at the WIKIRI Selva Viva/Centro Jambatu (Quito, Ecuador) and the butterflies (*E. isabella* = 4, *H. atthis* = 2, *H. erato* = 2, *H. ismenius* = 2, *H. melpomene aglaope* = 2, *H. melpomene plessen* = 2) were photographed at the Mariposas de Mindo–Butterfly Garden (Mindo, Ecuador). We also refer to recently published data on a UV reflective population of *O. sylvatica* (“Lita” morph) that was photographed in the wild (Yeager & Barnett, 2020).

To capture reflectance values across an ecologically relevant spectrum, we took calibrated photographs in both human‐visible (VIS = ~400–700 nm) and ultraviolet wavelengths (UV = ~300–400 nm), following methods outlined in Yeager and Barnett (2020). In short, we took all digital images using a tripod‐mounted, UV‐sensitive, full‐spectrum quartz converted Canon EOS 7D that was combined with a metal body NIKKOR EL 80‐mm lens. For human‐visible spectra, we fitted the lens with a Baader UV‐IR blocking filter (allowing transmission of 420–680 nm), and for the UV photographs, we fitted a Baader UV pass filter (allowing transmission of 320–380 nm). We photographed each subject in both human‐visible and UV wavelengths, under natural downwelling illumination that was representative of the covered canopy forests where both butterflies and frogs occur. All images were saved in RAW format and included a 10% and a 77% reflectance standard that allowed for color calibration and scaling.

### 2.2 Image processing

We used the MICA toolbox in ImageJ v1.52k to calibrate, align, and combine our paired VIS and UV photographs into a series of multispectral images (Schneider et al., 2012; Troscianko & Stevens, 2015). We used the 10% and 77% reflectance standards to standardize the images, and each of the photo pairs was aligned manually. We then manually selected regions of interest (ROIs), from each multispectral image, by selecting up to six of the strongest UV‐reflecting regions (UV+), and up to six similarly sized and shaped adjacent regions that did not reflect UV (UV‐). These ROIs were selected to assess the role of UV reflectance specifically rather than to represent all aspects of the color pattern.

**FIGURE 1:**
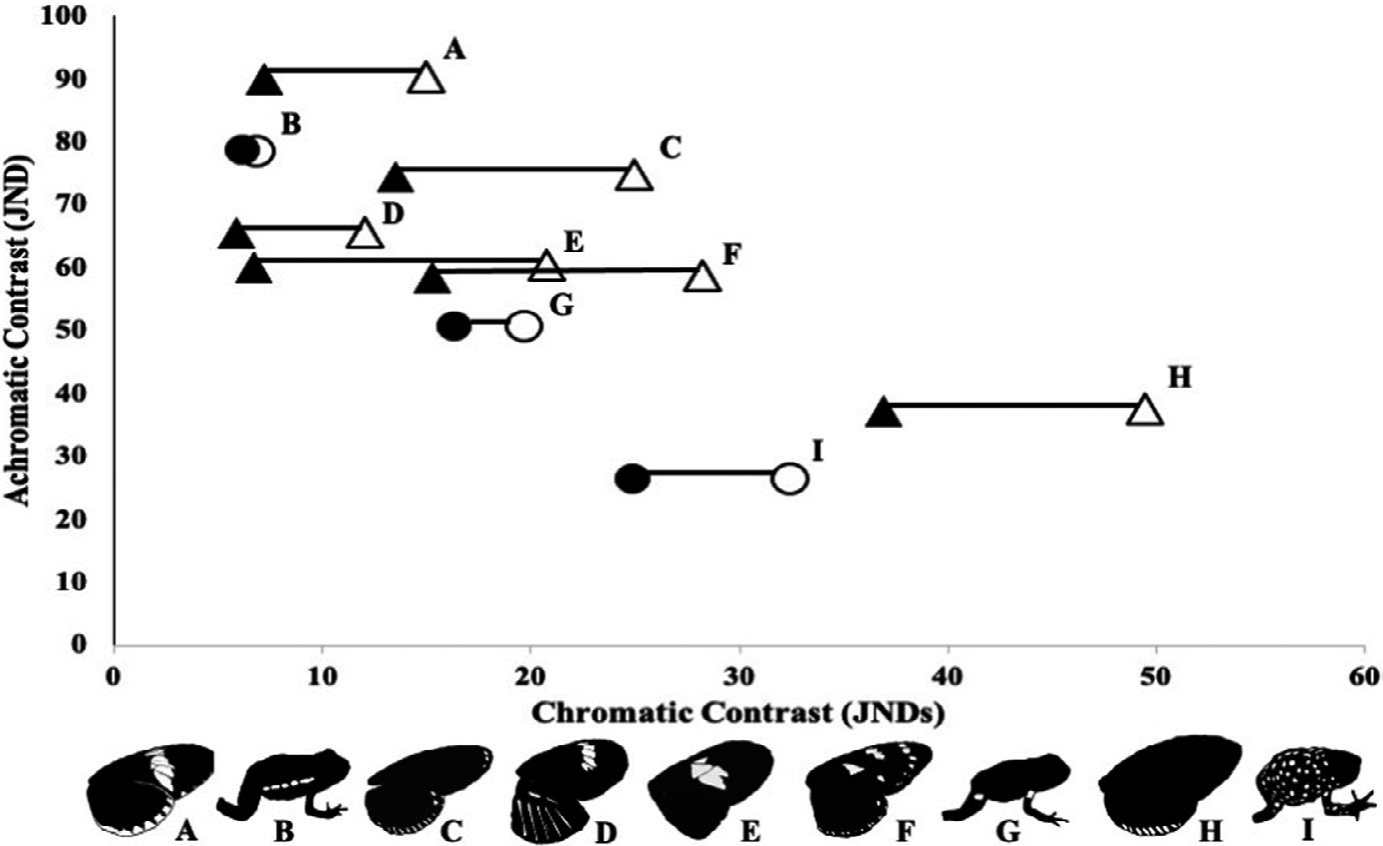
Contrast measured in chromatic and achromatic contrast mean “just noticeable differences” (JNDS) estimated for UV‐reflecting regions compared against adjacent non‐UV‐reflecting patches as viewed by UV‐sensitive models (open symbols) and VIS‐sensitive models (filled symbols). Triangles indicate butterfly species and circles poison frog species. In nearly all species, UV signals increase chromatic, but not achromatic contrast, but to differing degrees (see Table 1 for specific values and standard errors). Species in order of descending achromatic contrast: A = *Heliconius melpomene plessen*, B = *Epipedobates tricolor* Cielito, C = *Eueides isabella*, D = *Heliconius melpomene agalope*, E = *Heliconius erato*, *F* = *Heliconius atthis*, G—*Ameerega bilinguis*, H = *Heliconius ismenius*, and I = *Oophaga sylvatica* Lita (from Yeager & Barnett, 2020). UV‐reflecting color pattern regions are shown in white in the silhouette illustrations below

For the butterflies, we selected ROIs from the undersides of both the forewings and hindwings as these regions will be visible to both predators and conspecifics, and where most species had the greatest UV reflectance. In the frogs, the location of the UV‐reflecting regions was more variable but was limited to dorsal, lateral, and inguinal regions which would similarly be visible to avian predators. All ROIs were also chosen to avoid regions of specular reflectance (see Figure 1 for species‐specific UV reflection regions).

### 2.3 Visual modeling

To investigate how the presence of UV reflectance affects visual contrast between adjacent UV+ and UV− regions, we compared the responses of a UV‐sensitive visual model to those of a VIS‐sensitive visual model (Troscianko & Stevens, 2015; Yeager & Barnett, 2020). In order to control for all non‐UV‐sensitive components of visual perception, these models were both generated using the UV‐sensitive, tetrachromatic, vision of the Eurasian blue tit (*Cyanistes caeruleus*, Paridae). The blue tit has single‐cone λ_max_ of 573 nm (LWS), 508 nm (MWS), 413 nm (SWS), 372 nm (UVS), and double cones with λ_max_ of 565 nm (D) (Hart et al., 2000). The UV‐sensitive model included the LWS, MWS, SWS, and UVS cones spanning 300–700 nm, whereas the VIS‐sensitive model used the LWS, MWS, and SWS cones (excluding the UVS cone), to cover 400–700 nm. We also included the response of the D cone, which is not sensitive to UV light, in both visual models.

We converted each multispectral image into relative cone capture rates using the MICA toolbox in ImageJ v1.52k (Schneider et al., 2012; Troscianko & Stevens, 2015). Visual contrast was calculated as “just noticeable differences” (JNDs) using the receptor noise‐limited model (Vorobyev & Osorio, 1998). A JND of 1 represents the theoretical visual discrimination threshold below which two colors cannot be distinguished (Vorobyev & Osorio, 1998). Conversely, JNDs >3 are increasingly more easily discernable (Vorobyev & Osorio, 1998). We calculated chromatic (hue) contrast from the responses of the single cones and calculated achromatic (luminance) contrast from the response of the double cone (Hart et al., 2000; Vorobyev & Osorio, 1998). In both cases, we used Weber fractions of 0.05 (Hart et al., 2000; Troscianko & Stevens, 2015; Vorobyev & Osorio, 1998). We hypothesized that if UV reflectance is an important component of the signal, chromatic contrast would be perceivably higher in the UV‐sensitive model than is the VIS‐sensitive model (Yeager & Barnett, 2020).

## 3 RESULTS

We found that in all instances visual contrast between adjacent UV+ and UV− color patches was high, with JND values well above the conservative discrimination threshold (JND = 3, Table 1). However, when comparing between UV‐sensitive and VIS‐sensitive models, we found that although achromatic contrast was nearly identical between the two models, there were differences in chromatic contrast (Table 1). For all comparisons, chromatic contrast was higher in the UV‐sensitive model, although the magnitude of the effect varied by taxa. Moreover, when considering each species/subspecies individually, we found that the magnitude of the difference between UV and VIS models was very high for butterflies but comparatively low for frogs in both relative and absolute terms (Figure 1, Table 1).

**TABLE 1:** Chromatic and achromatic contrast from the VIS‐ and UV‐sensitive visual models (JND means ± *SE*), and the absolute and relative (%) difference in mean contrast between VIS and UV models (* *Oophaga sylvatica* (Lita locality) data from Yeager and Barnett (2020))

	Chromatic contrast			Achromatic contrast		
	VIS model	UV model	Difference (%)	VIS model	UV model	Difference (%)
Poison frogs						
*Ameerega bilinguis*	16.40 ± 4.24	19.71 ± 2.97	3.31 (20.18%)	50.63 ± 2.57	50.69 ± 2.59	0.06 (0.12%)
*Epipedobates tricolor* (Cielito morph)	6.21 ± 0.91	6.87 ± 1.29	0.66 (10.63%)	78.63 ± 4.81	78.55 ± 4.87	−0.08 (−0.10%)
*Oophaga sylvatica* (Lita morph) *	24.96 ± 5.05	32.39 ± 6.76	7.43 (29.77%)	26.22 ± 4.29	26.45 ± 4.30	0.23 (0.88%)
Heliconiinae butterflies						
*Eueides isabella*	13.47 ± 1.89	24.99 ± 0.73	11.52 (85.52%)	74.30 ± 1.97	74.71 ± 1.97	0.41 (0.55%)
*Heliconius atthis*	15.28 ± 3.94	28.25 ± 1.01	12.97 (84.88%)	58.26 ± 7.77	58.72 ± 7.92	0.46 (0.79%)
*Heliconius erato*	6.68 ± 2.98	20.74 ± 3.98	14.06 (210.48%)	59.99 ± 9.34	60.30 ± 9.37	0.31 (0.52%)
*Heliconius ismenius*	36.91 ± 3.48	49.50 ± 1.35	12.59 (34.11%)	37.21 ± 9.38	37.37 ± 9.42	0.16 (0.43%)
*Heliconius melpomene aglaope*	5.84 ± 4.52	12.06 ± 5.88	6.22 (106.51%)	65.30 ± 6.22	65.50 ± 6.28	0.20 (0.31%)
*Heliconius melpomene plessen*	7.18 ± 4.35	15.00 ± 2.68	7.82 (108.91%)	89.90 ± 4.49	90.19 ± 4.40	0.29 (0.32%)

## 4 DISCUSSION

We found that our sample of heliconiian butterflies and poison frogs all reflected detectable quantities of ultraviolet light. When comparing between VIS‐ and UV‐sensitive visual models, this UV reflectance had a negligible effect on achromatic contrast but did affect chromatic contrast, to varying degrees. The visual signals of heliconiian butterflies and poison frogs have both evolved under the influence of UV‐sensitive predators for the purpose of mitigating predation risk via aposematism. However, we found that UV reflectance from butterfly color patterns had a much greater effect on enhancing chromatic contrast, both in terms of absolute (change in JNDs) and in terms of proportional (percent increase due to the addition of UV) change, than was recorded from the color patterns of either of the poison frog species.

Many heliconiian butterflies have evolved highly contrasting signals that contain a significant amount of UV light. However, despite high contrast, and likely being visible to potential predators, UV reflectance does not appear to play an important role in predator aversion (Dell'Aglio et al., 2018; Finkbeiner et al., 2017). The most compelling selection‐based explanation for the evolution of UV+ signals comes from their potential use(s) for sexual selection in the genus *Heliconius*, where UV+ 3‐hydroxy‐DL‐kynurenine (3‐OHK) yellow pigments coincide with the duplication of UVS opsin genes (Briscoe et al., 2010). These signals have, therefore, co‐evolved with complex UV‐sensitive visual systems that allow heliconiian butterflies to tune into UV reflectance for both mate choice and species recognition (Briscoe et al., 2010; Bybee et al., 2012; Finkbeiner et al., 2014, 2017). Visual discrimination that potentially plays an important role in preventing intergeneric hybridization between mimetic *Heliconius* and *Eueides* butterflies (Finkbeiner et al., 2017). Indeed, all *Heliconius* species have duplicated UV coding opsin genes, and certain clades of *Heliconius* (such *as H. erato*, UV contrast shown in Figure 1e) can respond differently to different UV wavelengths. Yet even in these clades, such behavior appears limited to females, highlighting the role of sex‐specific selection in the evolutionary ecology of heliconiian UV signaling (Finkbeiner & Briscoe, 2020; McCulloch et al., 2016).

In comparison, UV reflectance in poison frogs appears to only have a small effect on pattern contrast, and its utility, if any, remains unknown (Yeager & Barnett, 2020). Although color can be an important intraspecific signal for poison frogs (Maan & Cummings, 2009; Yang et al., 2019), the lack of UV‐sensitive photoreceptors in the dendrobatid visual system means that it is unlikely that ultraviolet reflectance has evolved in response to mating preferences or intraspecific recognition. Importantly, however, visual perception has only be characterized for *O. pumilio* (Siddiqi et al., 2004), a species that lacks UV reflectance (Chaves‐Acuña et al., 2020; Maan & Cummings, 2009; Siddiqi et al., 2004; Summers et al., 1999). Therefore, although UV‐sensitive photoreceptors are unlikely, we cannot conclusively rule out the presence of UV‐sensitive vision in other dendrobatid species. Moreover, as strong UV reflectance does not appear to affect predation risk in artificial targets (Lyytinen et al., 2001) or heliconiian butterflies (Finkbeiner et al., 2017), it also seems improbable that the comparatively weak UV reflectance observed in poison frogs would be an important contribution to aposematic signals. Indeed, where UV reflectance has been reported in poison frogs pattern contrast remains high when UV is excluded, and other color pattern combinations that lack UV have both been found to result in greater visual contrast (Yeager & Barnett, 2020) and to be more likely to be avoided by avian predators (Lawrence & Noonan, 2018).

Maximizing visual contrast is not necessarily the goal of aposematic signals, and two patterns can be visually distinct (e.g., different combinations of colors) while being equally contrasting. However, by quantifying the contribution of UV to achromatic and chromatic contrast, our approach allows us to estimate the relative importance of these wavelengths to signal design. That said, it is important to note that the presence of UV reflectance within a color pattern does not equate to UV serving an explicit function; and depending on context UV reflectance could act as aposematism, camouflage, sexual signaling, thermoregulation, or protection from solar radiation (Umbers, 2013). Moreover, pigments and structural colors will interact with light beyond the wavelengths visible to observers, and reflectance characteristics outside of the visible range may evolve without direct selection. For example, selection for very high reflectance across 400–700 nm would very plausibly, as a by‐product, also produce significant reflectance in the near ultraviolet (350–400 nm) and near infrared (700–750 nm).

We cannot, therefore, definitively state a function for ultraviolet reflectance in poison frogs, if indeed there is a function. However, by directly comparing the characteristics of frog coloring to the well‐known UV signals of heliconiian butterflies, we can provide guidance and set potential expectations for future research on poison frogs. Firstly, we believe that it is important to characterize the visual systems of a greater diversity of dendrobatid species considering the impressive diversity of intra‐ and interspecific color patterns, where some species reflect UV and many well‐studied species apparently do not. Secondly, behavioral trials both with potential predators and conspecifics are needed to examine whether observers respond differently to UV+ and UV− signals under natural lighting conditions. More widely, we also believe that the role of UV reflectance in aposematic signaling deserves more attention, or perhaps publication bias against nonsignificant findings needs to be addressed. Finally, we add further evidence in support of previous cautions regarding the over interpretation of function in animal coloration, such as those reported for UV reflectance in bird coloring (Stevens & Cuthill, 2007), and specifically suggest that neutral evolutionary processes may be more common in shaping animal color patterns than currently acknowledged.

Here, we investigated the degree to which UV reflectance affected the visual contrast of conspicuous signals given the recent discovery of UV reflectance in aposematic poison frogs (Yeager & Barnett, 2020) which we compare to UV reflectance in aposematic butterflies. UV is known to play an important role in intraspecific communication in heliconiian butterflies, and we found that UV had a correspondingly large effect on increasing the chromatic contrast of butterfly coloration. Conversely, poison frogs are not known to perceive UV light, and UV reflectance had a comparatively small effect on signal contrast. These data support the notion that UV reflectance does not necessarily have a special role in aposematic signal design and has likely evolved neutrally in many poison frogs (Yeager & Barnett, 2020); however, much remains unknown.

## CONFLICT OF INTEREST

None declared.

## AUTHOR CONTRIBUTIONS

**Justin Yeager:** Conceptualization (equal); Data curation (lead); Formal analysis (equal); Funding acquisition (lead); Investigation (equal); Methodology (equal); Project administration (lead); Resources (lead); Writing‐original draft (equal); Writing‐review & editing (equal). **James Barnett:** Conceptualization (equal); Formal analysis (equal); Investigation (equal); Methodology (equal); Project administration (equal); Writing‐review & editing (equal).

## Data Availability

Data have been deposited in Dryad https://datadryad.org/stash/share/TTJXGEF6y2UlFRjTrl2HyFMX4Nzl3mDdekiGWZ5Dw7A.
